# Permissive Hypotension vs. Conventional Resuscitation in Patients With Trauma or Hemorrhagic Shock: A Review

**DOI:** 10.7759/cureus.16487

**Published:** 2021-07-19

**Authors:** Leah Woodward, Mohammed Alsabri

**Affiliations:** 1 Internal Medicine, Saba University School of Medicine, The Bottom, BES; 2 Pediatrics, Brookdale University Hospital Medical Center, Brooklyn, USA

**Keywords:** trauma, hemorrhage, permissive hypotension, resuscitation, shock

## Abstract

Trauma is one of the leading causes of death, with hemorrhage being one of the most preventable aspects. Aggressive fluid resuscitation protocols were implemented before their value was critically evaluated. Permissive hypotension limits blood loss while maintaining adequate perfusion and positively impacts outcomes in actively hemorrhaging trauma patients.

Peer-reviewed articles pertaining to the use of hypotensive resuscitation were identified and selected from a search of the PubMed database. Based on this, seven primary research articles were selected for evaluation. The articles were grouped based on their approach to hypotensive resuscitation. We focused on the safety and viability of hypotensive resuscitation, compared it to normotensive resuscitation, and compared mortality rates.

Our review shows that hypotensive resuscitation is safe and has a decreased mortality rate when compared to normotensive resuscitation in hemorrhagic shock patients. There is less blood loss, hemodilution, ischemia, and hypoxia in tissues. Additional research is required to determine the exact parameters that are most beneficial in different patient populations.

## Introduction and background

Trauma is the leading cause of death in patients younger than 44 in the United States, the fifth leading cause of death at any age, and makes up 38% of the surgical population [1]. Most trauma deaths occur within the first 24 hours following injury while more than 50% of those deaths occur within an hour of injury [2]. Of all the trauma-related deaths, hemorrhage is the most preventable if managed appropriately [3].

The management of trauma patients has evolved through the years. Crystalloid fluid resuscitation was first used in the 1950s and 1960s while massive blood transfusions began in the 1970s [1]. It was believed that rapid, large-volume fluid resuscitation was key in treating hypotensive shock in trauma patients, as organ ischemia and decreased perfusion decreased survival [4]. It was recommended that crystalloid fluid was replaced at a 3:1 ratio to the amount of blood lost. Therefore, aggressive fluid resuscitation became widely used before its value was critically evaluated and proven [1]. Rapid fluid infusions may restore lost volume; however, it inhibits normal hormonal responses within the body, which attempt to return homeostasis naturally [5]. This can have a detrimental effect on outcomes. Normal blood pressure was used as the goal to maintain adequate intravascular volume and organ perfusion. Many patients experienced side effects related to fluid overloads such as edema, hemodilution, and an inflammatory response along with metabolic and cellular changes, which can affect the pulmonary, cardiac, and coagulopathy systems [1]. One serious complication that is linked to hemorrhagic shock is the “lethal triad”: acidosis, hypothermia, and coagulopathy. The lethal triad occurs when tissues become hypoxic and rely on the anaerobic metabolism, which creates lactic acid. Acidosis and hypothermia both inhibit the clotting cascade thereby decreasing the body’s ability to clot and stop the hemorrhage [1]. Hypothermia can be easily rectified while more research is needed to correct acidosis without worsening coagulopathy. Point-of-care testing of the coagulation parameters allows resuscitation strategies to be more personalized to the individual patient. Massive transfusion protocols (MTP) are another way in which emergency departments have attempted to rapidly inhibit the progression of the lethal triad [6].

There is speculation that massive fluid infusion before the control of the hemorrhage is detrimental to patients. The goal of permissive hypotension, defined as a mean arterial pressure below normal, is to reduce blood loss, prevent disruption of formed clots, and decrease rebleed injuries [7]. Permissive hypotension would allow less fluid administration and a lower mean arterial pressure while still maintaining tissue oxygenation [7]. Damage control resuscitation includes permissive hypotension, restricting crystalloid fluid infusion, and the use of blood products that mirror whole blood. The intention of these interventions is to limit metabolic complications [1]. The optimal blood pressure for organ perfusion still has not been determined [8]. Resuscitation strategies are a balancing act between limiting hypotensive shock while preventing additional bleeding with an increase in blood pressure.

Massive fluid infusions also have a systemic cost on healthcare. Trauma patients who suffer serious hemorrhages present a significant resource burden on the healthcare system. In the United States, a patient with a penetrating or blunt trauma injury will have a longer hospital stay and higher per-diem costs [9]. Permissive hypotension has the potential to be more cost-effective while providing more efficient treatment with better outcomes.

Hypothesis and objectives

Trauma presents a heavy burden on patients and the healthcare system. It is hypothesized that permissive hypotension will significantly improve mortality and decrease complications associated with traumatic hemorrhagic shock.

## Review

Methods

The articles reviewed in this literature analysis were obtained from the PubMed database from which the most recent search was conducted on April 30, 2021. The keywords/Mesh terms used for the search strategy were: ((hypotensive resuscitation) AND Adult ) Sort by: Most Recent (" permissive hypotension "[All Fields] OR "hypotensive resuscitation"[All Fields] AND (”trauma [MeSH Terms] OR "shock [All Fields] OR “hemorrhagic shock [All Fields]) AND (“adult [All Fields] OR “adult [MeSH Terms] OR [All Fields] AND (“trauma “[MeSH Terms] OR "hypotensive resuscitation"[All Fields]) OR (""[All Fields] AND ""[All Fields]) OR “delayed resuscitation”OR ("hypotensive resuscitation”.

Inclusion Criteria

Primary articles (randomized controlled trials (RCTs)) and cohort studies that evaluated adult patients (age > 18) with penetrating or blunt traumatic injury and suspicion of hemorrhage and focusing on hypotensive resuscitation were chosen. Reference lists and bibliographies of eligible peer-reviewed articles also were searched for relevant material.

Exclusion Criteria

Secondary or tertiary articles, including review articles and meta-analyses, were excluded. Control trials involving animal subjects, case series, articles that were published before 1990, and non-English literature were excluded. Studies focused on the children population (age less than 18 years) were also excluded. After applying these exclusion criteria, five articles were used in this literature analysis.

Results

Selected articles were all primary research focusing on the effect of permissive hypotension on actively hemorrhaging trauma patients. Researchers used multiple methods and approaches in their analysis of the data, which will be detailed below. This section presents seven articles that outline protocols and compares them against current standards (Table 1). The first section will present five articles focusing on the benefits and comparison of permissive hypotension to current standards. For the final two articles, we compare past mortality rates to current mortality rates in trauma patients and discuss the changes within trauma protocols along with future directions.

**Table 1:** Summary of reviewed articles SBP: systolic blood pressure; MAP: mean arterial pressure; FFP: fresh frozen plasma

Author	Date of Publication	Study Design	Level of Evidence	Study Population	Therapy or Exposure	Outcome/ Result
Bickell, W.H. [4]	1994	Prospective trial	1	598 adult patients with penetrating torso injuries (diastolic BP < 90 mmHg prehospital)	Immediate (pre-hospital) vs. delayed (once in the operating room) fluid resuscitation	70% of patients who received delayed fluid resuscitation survived vs. 62% who received immediate fluid resuscitation. P=0.04 23% of delayed vs. 30% of immediate pts had complications. Length of hospitalization was shorter in the delayed group.
Carrick, M.M. [10]	2016	Randomized controlled trial	1	168 patients in hemorrhagic shock with penetrating injuries	Experimental arm – maintained a target MAP of 50 mmHg; Control arm – standard fluid resuscitation of MAP = 65 mmHg.	Pt’s were followed for 30 days. Pts in the experimental arm received less fluid. They did not have significantly different mortality rates at 24 hours or 30-days post-op.
Dutton, R. P. [11]	2002	Randomized control trial	1	110 patients in hemorrhagic shock	Target SBP > 100 mmHg (conventional) or target SBP of 70 mmHg (low). Fluid was titrated to this endpoint until hemostasis was achieved.	Titration of fluid did not affect mortality. There was an overall survival of 92.7% (4 deaths) in each group.
Morrison, C. A. [12]	2011	Randomized controlled trial	1	90 patients in hemorrhagic shock	Experimental arm – maintained a target MAP of 50 mmHg; Control arm – standard fluid resuscitation of MAP = 65 mmHg.	Pts were followed for 30 days. Pts in the experimental arm received fewer blood products and IV fluids. They had significantly lower mortality in the early postoperative period and non-significant lower mortality at 30 days.
Oyeniyi, B. T. [13]	2017	Retrospective	3	Records from a level 1 trauma center. 7080 patients (498 deaths) from 2005-2006 8767 patients (531 deaths) from 2012-2013	Analyze the temporal distribution of trauma-related deaths. Introduction of hemorrhage control interventions (a bleeding control bundle)	There was a reduction in hemorrhagic death rates, likely associated with a multimodal bleeding control bundle of care.
Schreiber, M. A. [3]	2015	Prospective randomized pilot trial	1	192 patients with a pre-hospital BP < 90 mmHg	Experimental group – 250 cc of fluid if they had no radial pulse or SBP <70 mmHg and an additional 250 cc to maintain radial pulse or SBP > 70mmHg. Control group – received 2L initially and fluid as needed to maintain SBP > 110mmHg	The control group received on average 1L less of IV fluid. Controlled resuscitation may have an early survival advantage in blunt trauma injuries, there was no difference among patients with penetrating trauma.
Stein, P. [14]	2017	Retrospective observational study	3	Compared two-time periods (2005-2007) and (2012-2014). All adult Pts with an injury severity score > 16 were included.	Changes in trauma management protocols: which included permissive hypotension, goal-directed coagulation management, etc.	Reduced incidence of massive transfusion and a reduction in transfusions of RBCs and FFP.

Effect of Permissive Hypotension

In previous decades, it was widely accepted that patients with active hemorrhage and hypotension due to trauma were infused with large amounts of fluid to replace lost volume. However, this view is changing. Before being implemented, the viability and safety of permissive hypotension in trauma patients must first be determined. Schreiber et al. sought to determine this [3]. Patients who presented with blunt or penetrating trauma and had a systolic blood pressure of ≤ 90 mmHg were randomized into two groups in the pre-hospital setting (patients were excluded if they had a severe head injury or Glasgow Coma Scale (GCS) of < 8). Patients in the standardized resuscitation group (SR) were given an initial 2L of fluid and then received fluid as needed to sustain a systolic blood pressure (SBP) of 110 mmHg. Patients in the controlled resuscitation group (CR) were given an initial 250 cc of fluid if their SBP was below 70 mmHg, or if they did not have a palpable radial pulse, they then received 250 cc as needed to sustain an SBP of 70 mmHg. Patients were followed for two hours after arriving at the hospital or until the hemorrhage was controlled. On average, the SR group was infused with 2.0 L (SD 1.4) of fluid while the CR group was infused with 1.0 L (SD 1.5), with a difference of 1.0 L (95% CI: 0.6-1.4). Significantly less IV fluid was given to the CR group in the pre-hospital setting and within the first two hours of arriving at the hospital, however, they did receive more packed red blood cells and blood products. At 24 hours, there was no significant difference between the amount of IV fluid, packed red blood cells, or blood products given to the two groups. The SR group received significantly more fluid in the prehospital setting, however, there was no significant difference between the two groups' vital signs, hemoglobin, or standard coagulation assays. The SR group had a decreased platelet count but was still within the normal range. The mortality rate at 24 hours was 5.2% and 14.7% for the CR and SR groups, respectively [adjusted odds ratio (aOR) 0.39 (95% CI: 0.12, 1.25)]. Blunt trauma patients in the CR group had decreased mortality when compared to the SR group. This was not demonstrated in patients with penetrating trauma. There was not a significant difference between the two groups regarding overall mortality.

Bickell et al. compared survival, length of hospital stay, and postop complications in patients who received aggressive fluid resuscitation versus hypotensive fluid resuscitation post-traumatic injury to the torso [4]. Patients who qualified were placed into two groups, starting with paramedics in the prehospital setting. All 598 patients were treated with the standard protocol; the difference being patients in the delayed-resuscitation group did not receive fluid after the insertion of IV catheters. Once patients arrived in the operating room, fluid and packed red blood cells were given, no distinction was made between the two groups. Analysis of categorical data was made via chi-square tests, and continuous data via Mann-Whitney U rank-sum test. P < 0.05 was considered significant. With delayed resuscitation (permissive hypotension), patients arrived at the hospital with significantly decreased systolic blood pressure and higher hemoglobin concentration and platelet count in addition to shorter prothrombin and partial thromboplastin times. There was no significant difference shown between the groups in relation to systemic arterial pH or venous serum bicarbonate concentration. There was no significant difference in the amount of packed red blood cells, fresh frozen plasma, platelets, and blood transfusions between the two groups once they reached surgery. The rate of fluid delivery was significantly higher in the immediate-resuscitation group (during surgery) in order to maintain a systolic blood pressure of 100 mmHg at 117 ± 126 ml/min versus 91 ± 80 ml/min in the delayed resuscitation group (p = 0.008). Survival within the delayed resuscitation group (70%) was significantly higher than the immediate resuscitation group (62%, p = 0.04). Length of hospitalization was increased in the immediate resuscitation group, however, there was no difference in the length of time in the ICU. There was a trend towards higher complications within the immediate resuscitation group, however, it was not significant (p = 0.08).

Dutton et al. assessed the mortality of 110 patients enrolled in a randomized controlled trial focused on patients in hemorrhagic shock who were resuscitated using two different fluid protocols [11]. Patients who were actively hemorrhaging and had an SBP of < 90 mmHg were randomized into two groups once they reached the trauma center. The first group's fluid was titrated to a “conventional” SBP of >100 mmHg while the second to a “low” SBP of 70 mmHg. Blood pressure was titrated using crystalloid or blood products as appropriate and the end of active bleeding was determined by the trauma surgeon and anesthesiologist. Statistical analyses were completed with a two-tailed comparison of independent variables with X² analysis and were considered statistically significant when p < 0.05. The SBP between the two groups was significant at 114 mmHg vs 100 mmHg (p < 0.001) while the duration of hemorrhage was not significantly different (2.97 +/- 1.75 hours vs. 2.57 +/- 1.46 hours, p=0.20). There were four deaths in each group, with overall survival of 92.7%.

Morrison et al. wanted to assess the 30-day morbidity and mortality of 90 patients enrolled in a prospective, randomized controlled trial focusing on hypotensive resuscitation [12]. Patients who had a systolic blood pressure of ≤ 90 mmHg, after presenting with a blunt or penetrating trauma, were randomly assigned to two groups once in the operating room. Patients were excluded if the presence of a traumatic brain injury was not ruled out. The experimental group had a target MAP of 50 mmHg (LMAP) while the control group had a target MAP of 65 mmHg (HMAP). All continuous variables were analyzed using Student’s t-tests and categorical data were analyzed using X² analysis (or Fisher’s exact test when n < 5). The 30-day mortality was compared using Kaplan-Meier curves and the Cox proportional hazards model. Morrison et al. found that there was no significant difference between the two groups in regard to baseline laboratory results or clinical scoring systems, except for the injury severity score, which showed a higher score in the HMAP group (25.1) vs. the LMAP (17.9, p=0.02) [12]. This indicates increased severity of the injury or more anatomic areas being involved in the injury. The total amount of blood products transfused was significantly higher in the HMAP group (2,898 mL) as opposed to the LMAP group (1,594 mL, p=0.03). While there was no significant difference in the amount of total IV fluids, estimated blood loss, or the actual MAP throughout surgery (64.4 mmHg vs. 68.5 mmHg, p=0.15). The overall survival between the two groups was not significantly different (10 vs. 13) in LMAP vs HMAP. However, the number of deaths within the first 24 hours was significantly decreased in the LMAP group (6 of 10) vs the HMAP group (12 of 13, p=0.03). Coagulopathic bleeding-related deaths within the first 24 hours were significantly higher in the HMAP group (7 of 10) vs the LMAP group (0 of 6, p=0.01). After 24 hours, coagulation in both groups was similar. Anemia and thrombocytopenia were also similar. There was no significant difference between creatinine or postop transfusions of packed red blood cells, fresh frozen plasma or platelets, as well as hematocrit or platelet levels. The HMAP group did have significantly higher prothrombin time (PT), partial thromboplastin time (PTT), and international normalized ratio (INR) vs the LMAP group. 

Carrick et al. focused on 30-day postop mortality for penetrating injuries in 168 patients [10]. This was a prospective, two-arm, intent-to-treat, randomized controlled clinical trial, which randomized patients on arrival to the operating room (OR )with an SBP of < 90 mmHg into two groups: a target MAP of 50 mmHg (LMAP) or a target MAP of 65 mmHg (HMAP). The study intended to recruit 271 patients, but early termination was recommended, as it was unlikely to reach a significant difference in 30-day mortality. Intraoperative MAPs between the two groups were not significantly different at 65.5 +/- 11.6 mmHg and 69.1 +/- 13.8 mmHg, p=0.07 despite the different target MAPs. The HMAP group received more norepinephrine, epinephrine, and total fluids (5200 ml vs 4125 ml). There was no significant difference in the estimated blood loss or urine output between the two groups and the total blood products given in the first 24 hours. There was no significant difference in 30-day mortality (18 vs 21 in LMAP and HMAP, respectively, p = 0.47) or within the first 24 hours (11 vs. 16 in the LMAP and HMAP, respectively, p = 0.27). There were no significant differences in postop complications except for acute renal injury (ARI), which was higher in the HMAP group (13% vs. 30%, p = 0.01).

Trauma Management Over Time

Stein et al. compared two time periods and wanted to demonstrate the effect changes to the trauma protocols had on patient outcomes [14]. All adult (over 16 years) trauma patients, who had an injury severity score (ISS) of ≥ 16 were included in the study. Changes to the trauma protocol were introduced from 2009-2012; therefore, the years considered for analysis were 2005-2007 and 2012-2014. The changes introduced to the trauma guidelines between these two time periods included a primary whole-body CT scan, a goal-directed transfusion protocol, early administration of tranexamic acid, restrictive fluid resuscitation, permissive hypotension, and damage control surgery. Data were taken from an internal database and put into a spreadsheet, along with the anesthetic records from the resuscitation in the emergency department and surgery (if required) until admission to the ICU. Categorical data were analyzed using the chi-squared and Fisher’s exact tests while numerical data were analyzed using the Mann-Whitney U-test. The trauma-associated severe hemorrhage (TASH) score is used to predict massive transfusions (≥ 10 units of RBC). The incidence of massive transfusion was not only significantly reduced in the 2012-2014 cohort; it was approximately 50% of the predicted TASH score (3.7% observed vs. 7.5% predicted) while the 2005-2007 rate was 12.4% (comparable to the predicted 12.1%). The adjusted odds ratio for the significantly reduced 2012-2014 cohort is 0.16, 95%CI: 0.06-0.42. Platelet transfusions between the two times did not differ while the red blood cell and fresh frozen plasma amounts were decreased in 2012-2014. There was a significant increase in the use of tranexamic acid and coagulation factor XIII in 2012-2014 while fibrinogen and the prothrombin complex concentrate were unchanged. The amount of resuscitation fluid used was decreased in 2012-2014, as well as the 24-hour mortality, total mortality, length of ICU stay, and days of ventilator support. Overall, time in the hospital did not change significantly.

Oyeniyi et al. compared deaths due to trauma and the factors that have changed over time [13]. These factors included the cause of the trauma, as well as treatment and the effect on patient outcomes. The years considered for analysis were 2005-2006 and 2012-2013. The changes introduced between these two time periods included early resuscitation procedures and the control of bleeding (ex. hemostatic dressings, tourniquets, minimizing crystalloid resuscitation, coagulation monitoring). Patients over 16 years, who were declared dead at the hospital were included in the study. Data were collected and the primary cause of death was determined. Records were from a level 1 trauma center; 7,080 patients (498 deaths) from 2005-2006 and 8,767 patients (531 deaths) from 2012-2013. Nine groups were established: 1 - head injury; 2 - hemorrhage: 3 - systemic infection/multiple organ failure: 4 - respiratory failure; 5 - cardiac arrest; 6 - comorbid (secondary disease significantly contributed to death); 7 - pulmonary embolism; 8 - other; 9 - unknown (not enough data to determine). Analysis was done with the Mann-Whitney rank sum and the chi-square test; p < 0.05 was considered significant. Between the two time periods, there were no differences in gender, race, ethnicity, or the median injury severity score (ISS); however, the median age did increase from 46 (28-67) to 53 (32-73); p < 0.01. Overall mortality (including patients who were dead on arrival at the hospital) decreased from 7.6% (95% CI: 6.9-8.2) to 5.8% (95% CI: 5.3-6.3), a decrease of 24%; while purely in-hospital mortality dropped from 6.6% (95% CI: 6.0-7.2) to 4.7% (95% CI: 4.2-5.1), which is a 30% decrease. There was a significant decrease in hemorrhage-related mortality between these two time periods, dropping from 36% to 25% (p < 0.01). In the first hour after injury in 2005-2006, the leading cause of death was hemorrhage (60.3%) then head injury (37.5%). This changed in 2012-2013 to head injuries (52.7%) being the leading cause, followed by hemorrhage (38%). Hemorrhages made up 50% of deaths while head injuries made up 47% in the first 8 hours. From eight to 48 hours, head injuries increased to make up 83% of the deaths while hemorrhage decreased to 17% of deaths. Death within eight hours of injury was heavily influenced by hemorrhage.

Discussion

Protocols surrounding the management of actively hemorrhaging trauma patients have evolved over the years as more data are evaluated. The safety of hypotensive resuscitation has been questioned due to the negative effects of decreased oxygen to organs and tissues [12]. However, the research presented in this analysis clearly lays out that permissive hypotension is safe and has the potential to be beneficial. Attempting to maintain normal blood pressure while a patient is actively hemorrhaging leads to the ‘lethal triad,” which includes hypothermia, academia, and coagulopathy [12].

Benefits of Permissive Hypotension

Once it was determined that there was a need for more research in the area of permissive hypotension resuscitation in trauma patients, Schreiber et al. determined that it was both viable and safe [3]. On average, controlled resuscitation used 1L of fluid less than normotensive resuscitation within the first two hours. However, there was no significant difference in lab values or overall mortality. Using less crystalloid fluid and more blood products is part of the damage control resuscitation protocol [3]. Many of the patients included in the Schreiber et al. study had an ISS < 15, which means they would have been minimally injured [3]. Minimally injured patients would not have needed fluid resuscitation for survival.

The Bickell et al. study was more specific, only including patients with traumatic torso injuries [4]. Delayed resuscitation had an increased survival rate and a decreased length of hospitalization. It was suggested that aggressive fluids before surgical intervention and hemorrhage control would increase blood loss, disrupt clots, and potentially cause a secondary hemorrhage [4].

One of the main differences between hypotensive resuscitation and normotensive resuscitation is coagulopathy as demonstrated by Morrison et al. [12]. The overall survival rate was not significantly different, however, the number of deaths due to coagulopathy bleeding in the first 24 hours after injury was significantly lower in the hypotensive group, as there were no deaths due to bleeding after surgical correction of the hemorrhage [12]. This demonstrates that early permissive hypotension is a key factor in the early mortality of trauma patients. The significance of this data may change, as this was an interim analysis of a clinical trial and there were only 90 patients in the analysis (increased risk for type 2 error). Additionally, significantly fewer blood products were used in the hypotensive group, and this did not have a negative effect on mortality or morbidity. Once again, this shows the potential to improve cost and allocate resources for other patients [12].

Limitations

In Morrison et al., two separate MAP targets were set for trauma patients [12]. Interestingly, patients in the lower group (50 mmHg) usually maintained a blood pressure higher than 50 mmHg on their own. This demonstrates that the body has its own response to low blood pressure, which may aid in clotting and natural hemostasis [12]. This is a potential area for future research.

This literature review is limited to a narrative review of articles in English after 1990 with adult populations. Additionally, articles were discovered through a search of PubMed; therefore, there is the potential that articles were missed.

Future directions

As trauma protocols change based on evidence-based medicine, the mortality in trauma patients has decreased over the years. Additionally, Stein et al. showed that there was a significant decrease in the amount of fluid given to trauma patients [14]. As this was a retrospective study, a causal relationship cannot be determined. There were multiple changes to the trauma protocol, and it is impossible to determine the effect of each one individually. A second retrospective study was also analyzed, and it had many of the same limitations regarding causal relationships. However, Oyeniyi et al. determined that overall mortality, as well as mortality due to hemorrhage, decreased over time [13]. Hemorrhage is a significant factor in mortality in the first eight hours post-trauma. These studies demonstrate the need for a multi-modal approach to trauma patient resuscitation. Future research could be done to determine the effect of each individual aspect of the multi-modal system. Additionally, which aspects can be applied to other patient populations needs to be identified.

## Conclusions

The key to hypotensive resuscitation is providing sufficient fluid to prevent cardiovascular collapse and to perfuse organs, without giving excessive amounts that can cause increased bleeding and wash out clots. Hypotensive resuscitation appears safe and is associated with a decreased mortality rate when compared to normotensive resuscitation. There is less blood loss, hemodilution, ischemia, and hypoxia in tissues. Additional research is required to determine the exact parameters that are most beneficial and in which patient populations.
